# Positioning Animal Welfare in the One Health Concept through Evaluation of an Animal Welfare Center in Skopje, Macedonia

**DOI:** 10.3389/fvets.2017.00238

**Published:** 2018-01-10

**Authors:** Miroslav Radeski, Helen O’Shea, Daniele De Meneghi, Vlatko Ilieski

**Affiliations:** ^1^Animal Welfare Center, Faculty of Veterinary Medicine, Saints Cyril and Methodius University of Skopje, Skopje, Macedonia; ^2^Department of Biological Sciences, Cork Institute of Technology, Cork, Ireland; ^3^Department of Veterinary Science, University of Turin, Turin, Italy

**Keywords:** animal welfare, center, One Health, evaluation, transdisciplinarity, impact, stakeholders

## Abstract

The Animal Welfare Center (AWC) in Macedonia was established in 2009. The objectives of the center are animal welfare (AW) education, research, raising public awareness of AW, and increasing cooperation between the stakeholders. One Health (OH) was not the major focus of the AWC work initially, but, rather, a focus that evolved recently. The objective of this study was to evaluate the AWC from the OH perspective as an example case for positioning the AW within the overall OH concept. Three types of evaluation were performed: (1) assessment of OH-ness, by quantitative measurement of the operational and infrastructural aspects of the AWC; (2) impact evaluation, by conducting quantitative surveys on stakeholders and students; and (3) transdisciplinary evaluation, using semi-quantitative evaluation of the links of cooperation between the AWC and the stakeholders in society by the custom designed CACA (Cooperation, Activities, Communication, and Agreement) scoring system. Results for the OH-ness of the AWC showed relatively high scores for OH thinking, planning and working and middle scores for OH learning and sharing dimensions, i.e., dominance of the operational over infrastructural aspects of the AWC. The impact evaluation of the AWC shows that familiarity with the OH concept among stakeholders was low (44% of the respondents). However, there was a commonality among stakeholder’s interest about AW and OH. According to the stakeholders’ and students’ opinions, the influence of AW on Animal, Environmental, and Human Health is relatively high (in the upper third of the 1–10 scale). The transdisciplinary evaluation of the AWC indicated the presence of transdisciplinarity work by the AWC, with a higher focus on the Universities and Research Institutions and some governmental institutions, and less linked with the Non-Governmental Organizations and Professional Associations (Chambers), e.g., the Veterinary Chamber in Macedonia. The evaluations conducted indicated that the AWC’s work is closely dedicated to improving animal, environmental, and human health and has a considerable OH role among the stakeholders in the society. This study describes the significant role and importance that AW has in OH.

## Introduction

Implementation of the existing standards, raising awareness, and developing risk assessment criteria for animal welfare (AW) is a high priority for the European Union. The research conducted among member countries of the World Organization for Animal Health (OIE) identifies education and training in AW as among the most pivotal tools for solving major welfare problems (1). This also reflects AW initiatives worldwide, at national and regional levels, such as the National reference center for AW in Italy^1^ and National reference laboratories for official control of feed, food, animal health, and welfare in Ireland^2^, focusing on developing guidelines and standards, prioritizing welfare specific issues, raising awareness, implementation of EU legislation, strengthening capacities, supporting and conducting AW research, providing education and training, international cooperation and consultation practices at different levels. These were the main drivers and principles for initiating the work of the Animal Welfare Center (AWC) in the Republic of Macedonia.

The AWC was established in March, 2009 as part of the Faculty of Veterinary Medicine at the Ss. Cyril and Methodius University in Skopje, Macedonia (2). Besides the permanent staff, the AWC is also a merging point for experts of different fields from both within and outside the Faculty for AW-relevant issues on a national level. In April, 2010 the AWC signed a contract for cooperation with the competent authority—the State Veterinary Office. Besides formal recognition, the signed contract with the Macedonian government gave authorization and responsibility to the AWC for the provision of professional training and strengthening capacities for the veterinary authorities and conduction of vocational training and certification for different professionals where AW could be impaired. To date, the AWC has had extensive activities in relation to AW. The AWC has developed and implemented an AW course for undergraduate veterinary students. In addition, several workshops and projects have been held where the AWC was an integral part considering implementation of EU Directive 2010/63 for protection of animals used for scientific purposes and exploration of alternative techniques, the 3Rs Concept (Replacement, Reduction, and Refinement) (3). Likewise, AWC developed training courses for stakeholders in line with the EU directives (4–7). Regarding AW research, several studies were conducted which related to animal slaughtering, transport, animal behavior, etc. On a national level, the most important studies were the welfare assessment of poultry and dairy farms for the first time in Macedonia (8, 9). AW concerns about ambient conditions and air quality in the poultry and pig farms in the country were also raised. Later, this led to the assignment of the accreditation certificate for measuring air and noise emissions from housed farm animals (MKTC CEN/TS 15675:2009; MKS ISO 1996-2:2010) in the laboratory.

One Health (OH) is a term that captures integrative approaches to health and emphasizes the commonalities of human, animal, plant, and environmental health (10, 11). The strong link between AW and animal health, human health, and environment is evident (12, 13). Previous studies recognized the importance of AW to animal health, where animal health is as a crucial part of AW or even going to the extent, outlined by some authors, where animal health is the only explanation of AW (14). Taking care of AW and implementation of these standards is contributing to the reduction of the environmental impact from animal farms (12, 15), i.e., environmental health. Likewise, food safety and antimicrobial resistance are primary factors for human health that can be regulated and influenced by AW standards (13). The review by de Passillé and Rushen (12) suggests that improving AW in farms will reduce stress-induced immunosuppression, the incidence of infectious diseases on farms and the shedding of human pathogens by farm animals, antibiotic use and antibiotic resistance, and the environmental impact from the farm animals. Recently, a “One Welfare” platform for improving human and AW was presented by Pinillos et al. (16), where the interconnections between AW, human well-being, and environment are recognized. All of this implies that AW considerably impacts on OH. However, empirical and practical examples of these theoretical presumptions concerning the link between AW initiatives and OH are lacking.

Obviously, OH was not the major focus of the AWC work from the onset. However, if retrospectively analyzed, the activities of the AWC are in line with the possible links between AW and OH. Therefore, the objective of this study was to evaluate the AWC in Macedonia as AW initiative from the OH perspective, and use this case as a model for determining the links and relations of AW within the overall OH concept (human, animal, and environmental health), a model that could be possibly applied to evaluate other AWCs. The intention of this study was neither to present the AWC and its OH approach nor to describe the working areas of this single AW initiative. Ultimately, this study describes the role and importance of AW in general to OH by using the AWC as an example case.

## Materials and Methods

From the working continuum of the AWC, the evaluation in this study was limited to one extracted timeframe. The AWC work was observed and evaluated from its commencement to the end of 2016, i.e., almost 7 years. The evaluation was conducted in the last 3 months of 2016. Three types of evaluations on the AWC work from an OH perspective were performed: assessment of OH-ness, impact evaluation, and transdisciplinary evaluation. For each evaluation, different evaluation methods, approaches, and metrics were used.

### AWC Assessment of OH-Ness

For the quantitative measurement of the operational and infrastructural aspects of OH-ness of the AWC, the proposed methodology by the COST Action TD1404, Network for Evaluation of One Health (NEOH)^3^ for Assessment of OH-ness presented in “A Handbook for evaluation of one health,” Chapter 3—A One Health Evaluation Framework, Draft version from November 2016 was used. The assessment was performed by the staff permanently involved in the AWC from the beginning, i.e., the head and deputy of the AWC, who are experts in AW and experience in AW initiatives on national and regional levels. The given scores for each question and parameters requested in the assessment tools (S1_OH-ness Scoring of the AWC in Supplementary Material) represents the evaluation of the AWC as an OH initiative, not only as an AW initiative. These scores were the consensus of the assessors reached by taking the mean score from the scores given in the separate performed assessments. The assessment was performed for the following OH dimensions: thinking, planning, working (transdisciplinarity and leadership), learning, and sharing. The holistic approach of OH-ness was defined as a combination of the previous mentioned assessments into a One Health Index (OHI). This index is visually presented as a spider diagram of pentagonal structure and calculated according to surface of the pentagon defined by the enclosed lines that are connecting the points—assessment scores for different dimensions (from 0 to 1) (10), for details see S2_One Health Index and One Health Ratio in Supplementary Material. Precisely, the following equation was used for calculating the OHI:

(1) $$\text{OHI}=\frac{\text{sin}\frac{2\pi }{5}}{2}*\{(\text{ScP}*\text{ScT})+(\text{ScL}*\text{ScP})+(\text{ScS}*\text{ScL})+(\text{ScTD}*\text{ScS})+(\text{ScT}*\text{ScTD})\}$$

where ScP is the score obtained in OH planning, ScT is the score from OH thinking, ScL is the score obtained in learning infrastructure, ScS is the score from sharing infrastructure, and ScTD is the score from transdisciplinarity and leadership. In the described model, the range of OHI was from 0 to 2.37.

In addition, for presenting the balance between “operation” and “infrastructure” of the initiative, the One Health Ratio (OHR) was calculated. The operational aspects involved OH thinking and planning, while the infrastructure was constructed from OH learning and sharing. Transdisciplinarity and leadership were considered as evenly important for both operation and infrastructure. Therefore, this score was considered as a fixed point for the diagonal that divides the pentagon into two structures (operation and infrastructure). Thus, for calculating the OHR, the ratio between surfaces of the two defined quadrilaterals was calculated using the equation:

(2) $$\text{OHR}=\frac{\text{OH}{\text{I}}_{\text{operation}}}{\text{OH}{\text{I}}_{\text{infrastructure}}}=\frac{(\text{ScT}*\text{ScP})+(\text{ScTD}*\text{ScT})+\frac{\text{Sc}{\text{P}}^{2}}{2}}{(\text{ScS}*\text{ScL})+(\text{ScTD}*\text{ScS})+\frac{\text{Sc}{\text{L}}^{2}}{2}}.$$

### Impact Evaluation of AWC on OH

A quantitative survey was performed for determining the impact of the AWC work on OH. The target groups for this survey were different stakeholders, grouped according to whether they did or did not have cooperation with the AWC during the time period that this evaluation was focused on. The stakeholders included in this survey were categorized in six main categories: Farmers, Food Industry; Non-Governmental Organizations (NGOs); Academia; Governmental institutions; and Veterinary chamber. In addition, the veterinary students who took and did not take the AW course during their undergraduate studies were also involved. The survey was conducted by using a custom developed questionnaire, divided into four main sections: a general section for AW and OH; a section for respondents who had cooperation with AWC; a section for respondents who did not have cooperation with AWC; and a personal data section. The 25 questions in the questionnaire were different types, i.e., rating scales (from 1—minimum to 10—maximum), multiple choice, dichotomous, and open-ended questions (see S3_Questionnaire Form in Supplementary Material). To avoid any misunderstanding in terminology, the OH concept in the questionnaire was presented by setting questions directly focused on human, animal, and environmental health. Before collecting the data, the questionnaire was validated by 10 respondents (students and teachers at the veterinary faculty) giving feedback for improvement and polishing the final version of the questionnaire. The answers collected during the validation of the questionnaire were not part of the data collection process and were used only for the improvement of the questionnaire. Following this, the questionnaire was distributed to the respondents personally or electronically, and collected after completion.

The data collected from the questionnaire were analyzed using descriptive statistics, i.e., medians, ranges and 25 and 75% quartiles (Q1 and Q3) for the rating scale questions and frequencies of categorical and dichotomous variables. The grouping variables were based on the cooperation with the AWC, stakeholders’ categories, and student’s participation in AW course. Cross tabulation between different variables (questions) from the questionnaire was performed for presenting the link between AWC and OH. The correlation between self-graded knowledge about AW and the opinion of the respondents on the level of influence of AW to the human, animal, and environmental health was tested by using Spearman Rank Order test. Likewise, the differences between groups considering OH were tested by Mann–Whitney test and Fisher’s exact test or by Kruskal–Wallis ANOVA, setting the level of statistical significance at *P* < 0.05. The data analysis was performed by using STATISTICA 8.0 (StatSoft Inc., Tulsa, OK, USA) software.

### Transdisciplinary Evaluation of AWC

The transdisciplinary work of the AWC, i.e., the work that transcended academia and involved cooperation with different stakeholders in society (17), was evaluated by using custom designed semi-quantitative evaluation. The evaluation process was conducted in three main phases: identification; data collection; scoring and modeling. In the first phase, the relevant stakeholders were identified. Emphasis was given in identifying existing stakeholders for whom OH was specifically within their interest or their work was primarily related to human, animal, and/or environmental health. The identification procedure was performed by classifying the stakeholders into six major groups: Universities and Research Organizations; Animal farms; Government; the Food industry; Chambers; and NGOs. The organizations and institutions (actors) within each group were selected by searching the Macedonian databases of registered organizations/institutions in the Central Registry of the Republic of Macedonia; the Macedonian Government; the NGO sector database and Google search (by sectors in Macedonia). For each actor identified, the scope of work, mission, and objectives were reviewed. From the final pool of existing stakeholders, the actors who have a direct relationship to human, animal, or environmental health in Macedonian society were selected. The second phase—data collection, consisted of summarizing all realized projects, initiatives and activities by the AWC during the evaluation period using the documentation in the AWC archive. Later, this was supplemented by interviewing the AWC head and deputy regarding the AWC work and ongoing collaborations. All findings were entered into a matrix with information for each actor about the type of cooperation, the number of realized activities, the communication frequency, and the presence of formal agreement with AWC.

The last phase of this evaluation consisted of quantifying the links of cooperation between the AWC and the identified actors and stakeholders. This was carried out by developing a custom designed scoring system, abbreviated as CACA, based on four main pillars: Cooperation; Activities; Communication and Agreement. Each pillar has an equal contribution (25 points) with a final score of cooperation, giving a maximum of 100 points. Within each pillar there were different descriptors, bearing corresponding weights, depending of the level of contribution in the pillar. The level of contribution for different descriptors was developed by equalizing the different descriptors within one pillar in relation to the evaluation period of the AWC (almost 7 years). Thus, for the Cooperation pillar, seven descriptors were used, where the contribution level was determined by considering the strength of cooperation, starting with “Participation in the decision body,” indicating very strong cooperation, i.e., a contribution level of 100%. More than three “Project implementations” between AWC and the Actor within the evaluation period is considered as a strong cooperation, almost as strong as “Participation in the decision body.” Therefore, the contribution level of “Project Implementation” was 30%. Cooperation in “Research” is close to the “Project implementation” and was positioned in the middle between “Expertise” and “Project implementation,” whereas the three “Expertise” engagements within the evaluation period were considered was having almost the same strength of cooperation as “Project implementation,” i.e., the contribution level for “Research” was 20% and for “Expertise” 10%, of the overall score of this pillar. Three “Education and Trainings” within the evaluation period were considered almost equal to “Expertise” leading to the contribution level for “Education and Trainings” of 3% and so on until the contribution level for “Meeting” of 0.2%. The same approach for determining the contribution level of different descriptors was used for the descriptors for the Communication and Agreement pillars. For the “Activities” pillar, it was considered that if there were at least 10 joint activities between the AWC and the specific actor within the evaluation period, then the maximum score for this pillar should be given, i.e., each joint activity has a contribution level of 10%. Maximum score for each pillar was 25 and the weight for each descriptor was calculated from the contribution level as a percentage of this maximum score. If the score for the particular pillar is >25 then the given score for the pillar was 25. The pillars, their descriptors, and appropriate weights of the CACA scoring system are presented in detail in Table 1.

**Table 1:** Pillars, descriptors, and weights of the CACA scoring system for cooperation between Animal Welfare Center and the society’s actors/stakeholders.

Pillar	Cooperation			Activities			Communication			Agreement		
Pillar’s description	Type of realized cooperation			Number of realized activities			Frequency of communication			Formally signed agreement with the representatives within the actor		
				
	Descriptor	C%	W	Descriptor	C%	W	Descriptor	C%	W	Descriptor	C%	W
	Meeting	0.2	0.05	One joint activity	10.0	2.50	Once in several years	6.0	1.50	No signed agreement	0.0	0.00
	Workshop	1.0	0.25				Yearly	12.0	3.00	Agreement with ≤25% of the actors	25.0	6.25
	Education and trainings	3.0	0.75				Once in 6 months	25.0	6.25	Agreement with 26–50% of the actors	50.0	12.50
	Expertise	10.0	2.50				Quarterly	50.0	12.50	Agreement with 51–75% of the actors	75.0	18.75
	Research	20.0	5.00				Monthly	100.0	25.00	Agreement with 76–100% of the actors	100.0	25.00
	Project implementation	30.0	7.50									
	Decision body participation	100.0	25.00									

*C, level of contribution of the descriptor within the pillar, in percentages; W, weight of the descriptor within the pillar*.

The calculations for quantifying the links of cooperation represented with one score for the cooperation between AWC and the analyzed actor were carried out using Eq. 3:

(3) $${\text{S}}_{\text{CACA}}=\sum_{{\text{C}}_{\text{d}}=1}^{7}{\text{C}}_{\text{d}}+{\text{A}}_{\text{n}}\times 2.50+{\text{C}}_{\text{o}}+{\text{A}}_{\text{g}}$$

where the total score for cooperation between AWC and the actor (S_CACA_) represents the sum of the sum of weights of seven descriptors from Cooperation (C_d_), number of joint Activities (A_n_), Communication (C_o_), and Agreement (A_g_). For example, if one actor has two education trainings, one workshop and one project implementation, realized three joint activities with the AWC, communicates with the AWC on a quarterly basis and the AWC has an agreement with less than 25% of the members of this actor than the overall score will be: (2 × 0.75 + 1 × 0.25 + 1 × 7.50) + 3 × 2.50 + 12.50 + 6.25 = 35.50. Finalized on the scores for cooperation between AWC and the existing actors, the model of transdisciplinarity of the AWC was created, presenting the strengths of cooperation and positioning the AWC within society from the OH perspective.

## Results

### OH-Ness

The OH-ness of the AWC revealed different scores for each dimension following the questions and parameters within the dimensions. Detailed scoring results of the AWC with the complete evaluation for the five dimensions of OH-ness are presented in the S1_OH-ness Scoring of the AWC in Supplementary Material. The score for the OH Thinking dimension of the AWC was 0.79, with the highest scores of 1.00 for: the variety of the number of dimensions and scales that reflect and integrated approach to health; thinking at structural level considering the features of the system which are targeted by the AWC; and considering the capability of AWC to target different elements of the chain of events in relation to a problem. The lowest score (0.40) within this dimension was regarding the wellness of the initiative (AWC) matching the environment. The score for OH Planning of AWC was 0.75, where half of the stakeholders within the tasks returned the highest score and the other half were mid scored. The OH Working dimension (transdisciplinarity and leadership) of the AWC was scored with 0.70 points. The scores within this dimension ranged from 1.00 for the societal aspect and broadness and 0.59 for the integration of the AWC. The lowest scores of the AWC were for the OH Learning and OH Sharing dimensions of 0.47 and 0.46, respectively. In the OH Learning dimension, the highest score (0.75) was for the learning on individual and organizational levels, while the lowest score (0.13) was for the support of the general environment for adaptive and transformative learning. The highest score (1.00) in the OH Sharing dimension was about the usage of information in learning and the lowest 0 scores were given to the sharing resources and data accessibility.

**Figure 1:**
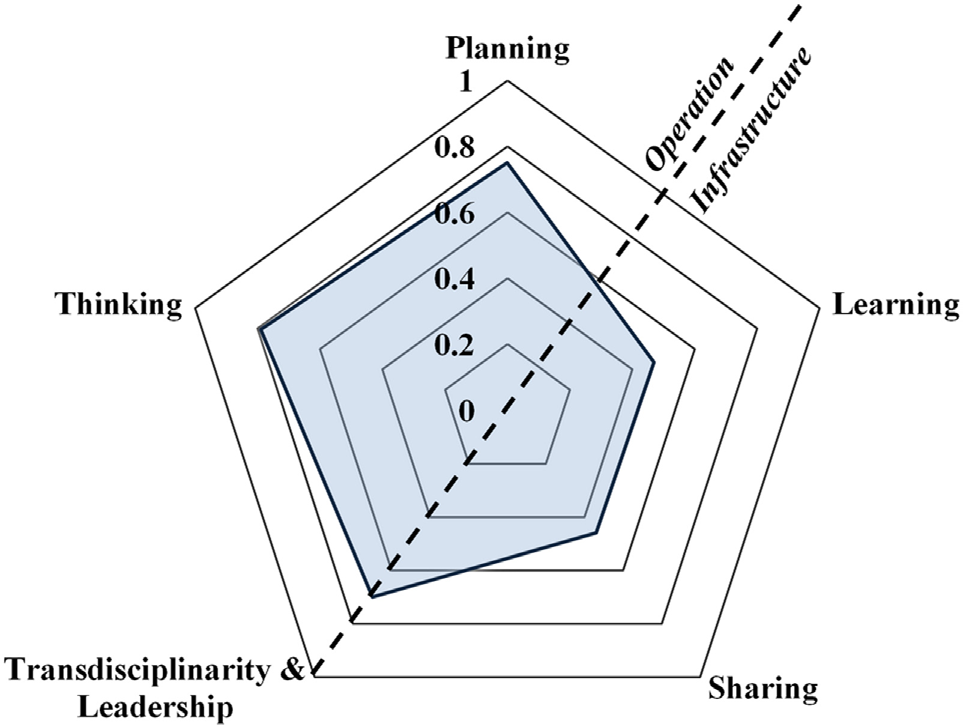
Spider diagram based on the scores (from 0 to 1, solid lines) of the five dimensions for the One Health Index of the Animal Welfare Center (AWC) (the transparent blue structure) from the One Health perspective. The dashed line represents the division of the diagram into Operation and Infrastructure for representing the One Health Ratio of the AWC.

Following analysis of the scores’ dimensions and using Eq. 1, the OH Index of the AWC from the OH perspective was 0.97. By using Eq. 2, the score for the Operation of the AWC was 0.68, while the score for the Infrastructure of the AWC was 0.31, leading to the OH Ratio of 2.20. The overall appearance of the OH-ness of the AWC spider diagram defined by the OH Index and Ratio is presented in Figure 1.

### Impact Evaluation

The survey was completed by 36 representatives (85% response rate) from different stakeholders: Government Institutions, such as the Food and Veterinary Agency, Ministry of Environment and Physical Planning, Local government, and other governmental sectors; Academia, i.e., universities and research institutions; NGOs; animal farmers; food processing industry; and Veterinary Chamber (Table 2). Geographically, 63% of the representatives were from Skopje—the country’s capital, and, regarding gender, 39% were female respondents. From the stakeholder’s representatives, 53% stated that currently or in the past have established cooperation with the AWC. The student’s survey included 30 veterinary undergraduate students (100% response rate), 15 of these (50%) were students who did take the AW course during their studies and rest of the respondents did not take this course. The geographical and gender structure of the students, respondents in the survey, was 73% from Skopje and 50% were females, respectively.

**Table 2:** Scores, presented as median, range, and 25–75% quartiles (Q1–Q3), given by the stakeholders regarding the influence level of Animal Welfare on Human (HH), Animal (AH) and Environmental (EH) Health, considering their cooperation with the Animal Welfare Center (AWC).

Stakeholder (*N*)	Cooperation with AWC							No Cooperation with AWC						
	*n*	HH		AH		EH		*n*	HH		AH		EH	
		Median Score	Range (Q1–Q3)	Median score	Range (Q1–Q3)	Median score	Range (Q1–Q3)		Median score	Range (Q1–Q3)	Median score	Range (Q1–Q3)	Median score	Range (Q1–Q3)
Non-Governmental Organizations (6)	0							6	9.50	7–10 (8–10)	10	8–10 (10–10)	10	8–10 (8–10)
Farmers (5)	4	7	7–8 (7–7.5)	9.5	9–10 (9–10)	7.5	7–10 (7–9)	1	10		10		10	
Academia (8)	8	7	5–10 (5.5–8.5)	10	8–10 (9.5–10)	8	4–10 (6–10)	0						
Governmental institutions (13)	5	7	5–10 (7–9)	9*	9–10 (9–10)	9	5–10 (7–10)	8	7	5–8 (6–7)	9*	7–9 (7.5–9)	7.50	5–9 (6.5–8.5)
Chambers (2)	1	7		9		7		1	8		9		8	
Food industry (2)	1	9		9		6		1	8		8		8	

*Significant difference (*P* < 0.05) between groups within the Governmental institutions considering AWC cooperation.

**Figure 2:**
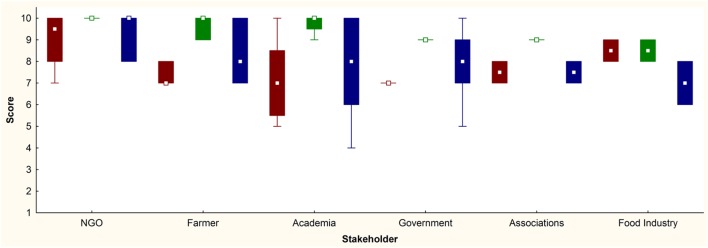
Stakeholders’ (Non-Governmental Organizations, *n* = 6; farmers, *n* = 5; academia, *n* = 8; government, *n* = 13; associations, *n* = 2; food industry, *n* = 2) opinions regarding the influence level (in scores on the *Y* axis) of Animal Welfare on Human (red), Animal (green), and Environmental (blue) Health. Median □; 25–75%, box; non-outlier range, whisker.

The overall concept of OH was familiar to 44% of stakeholder’s representatives. The number of respondents who had cooperation with the AWC was significantly higher (63%) than those who did not have cooperation (24%), regarding familiarity with the OH concept. On a scale of 1–10, the level of influence of AW on human, animal, and environmental health, the stakeholders responded with the median score of 7 (range 5–10, Q1 = 7 and Q3 = 9), 9 (range 7–10, Q1 = 9 and Q3 = 10), and 8 (range 4–10, Q1 = 7 and Q3 = 10), respectively. Detailed results concerning the opinion of different stakeholders regarding the influence of AW on OH are presented in Figure 2. The introduction of the grouping variable for cooperation with AWC revealed no significant difference between groups when considering the influence of AW on human, animal, and environmental health. However, there were various scores among different stakeholders regarding this issue, descriptively presented in Table 2. Due to the small sample sizes of different stakeholder groups, the only comparison considering AWC cooperation was carried out between Governmental institutions, revealing significant differences for the opinion about the influence of AW on Animal Health (Table 2). The correlations between the stakeholder’s self-graded knowledge of AW and human, animal, and environmental health were 0.31, 0.29, and 0.27, respectively.

**Figure 3:**
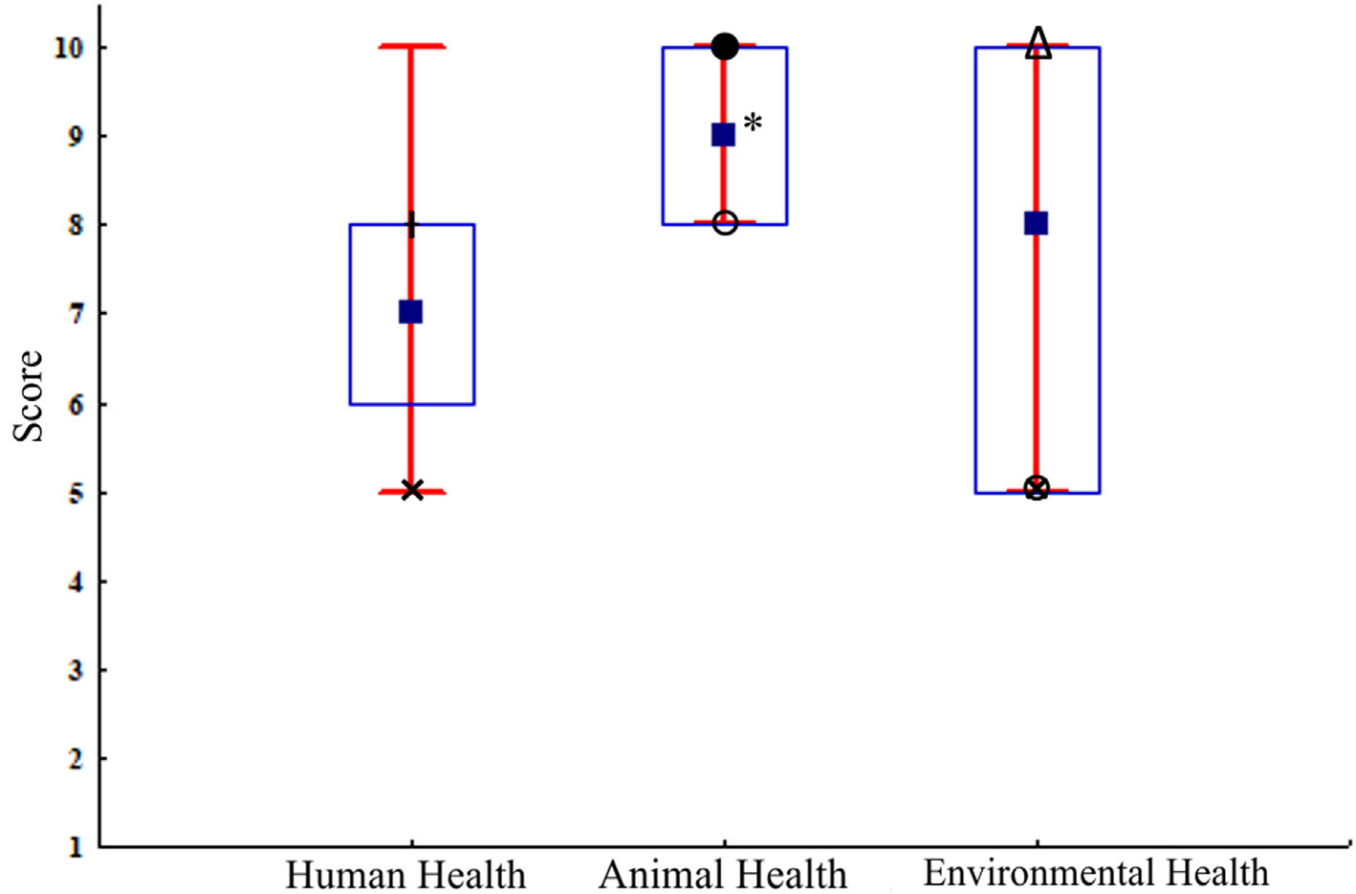
Impact of the Animal Welfare Center (AWC) on human, animal, and environmental health on a score scale from 1 to 10 according to the respondents who had cooperation with the AWC (*n* = 19). Median □; 25–75%, box; non-outlier range, whisker. *P < 0.05. Lowest and highest median scores for the three types of health given by the stakeholder’s groups (× Food industry, I Farmers, ○ Veterinary Chamber, ● Academia, and Δ Governmental institutions).

**Figure 4:**
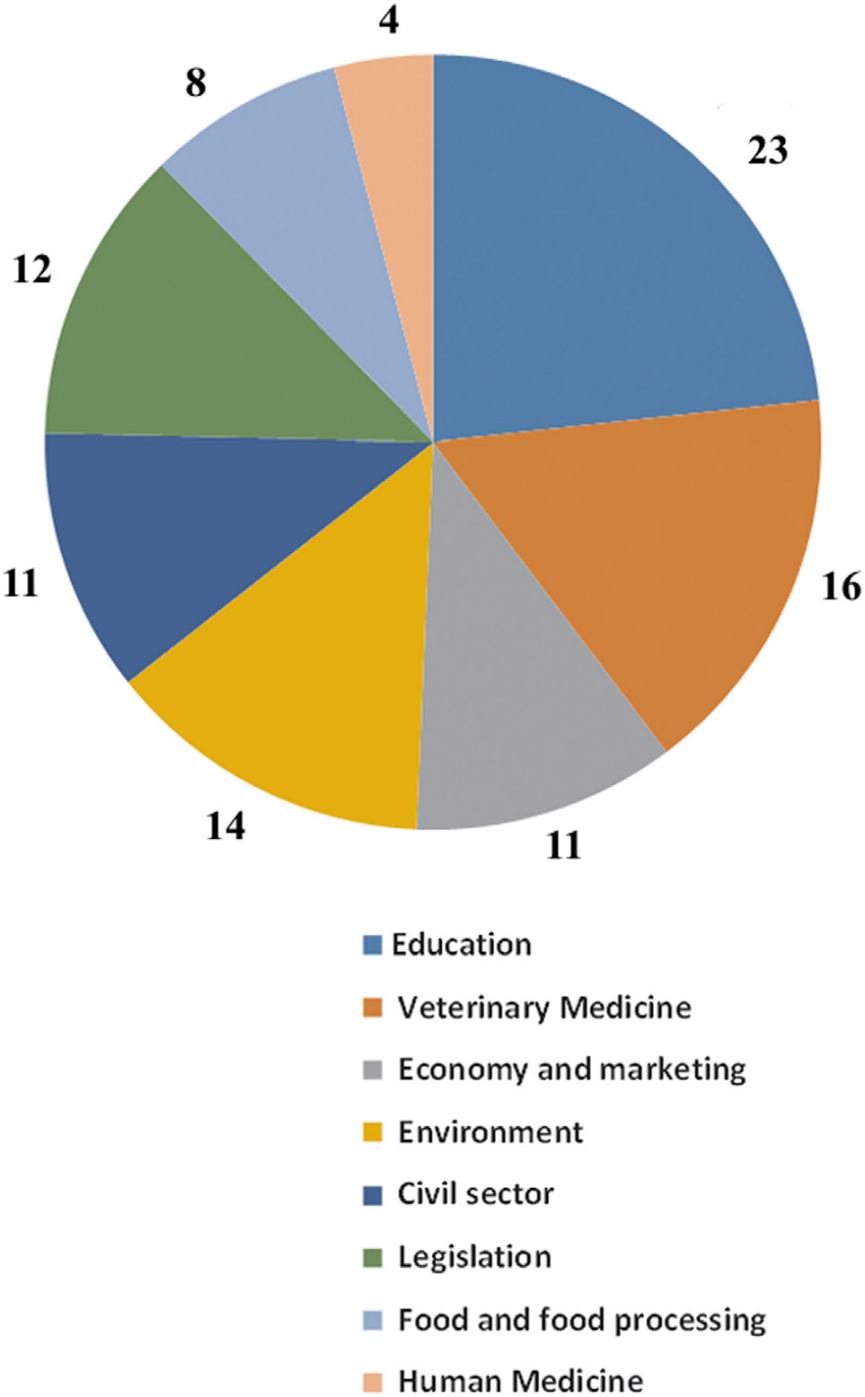
Stakeholder’s opinions, from the respondents who had cooperation with the Animal Welfare Center (AWC) (*n* = 19), regarding the areas where the AWC should expand its activities for improving human, animal, and environmental health (the numbers represent percentages from the responses for all areas).

The stakeholder’s representatives who had cooperation with AWC graded the cooperation with average grade of 4.16 ± 0.76 (on a scale from 1 to 5). This group of respondents scored the impact of the AWC on human, animal, and environmental health, where the impact on Animal Health was significantly higher in comparison with Environmental and Human Health (Figure 3). Lowest and highest AWC impact median scores for different types of health considering the groups of stakeholders are presented in Figure 3. All respondents consider that by cooperating with the AWC they are contributing to improving human, animal, and environmental health. The respondents who have cooperated with the AWC stressed that the AWC should expand its activities almost equally in all areas in order to improve the OH (Figure 4). The majority of the respondents who did not have cooperation with the AWC (85% of the respondents) believe that if they cooperate with the AWC they could contribute to improving human, animal, and environmental health. Summarizing the other answers from this group of respondents and the other open-ended questions in the survey, the most frequent statement given as a major remark or as a suggestion for higher involvement was the need for better promotion of the AWC’s activities.

**Figure 5:**
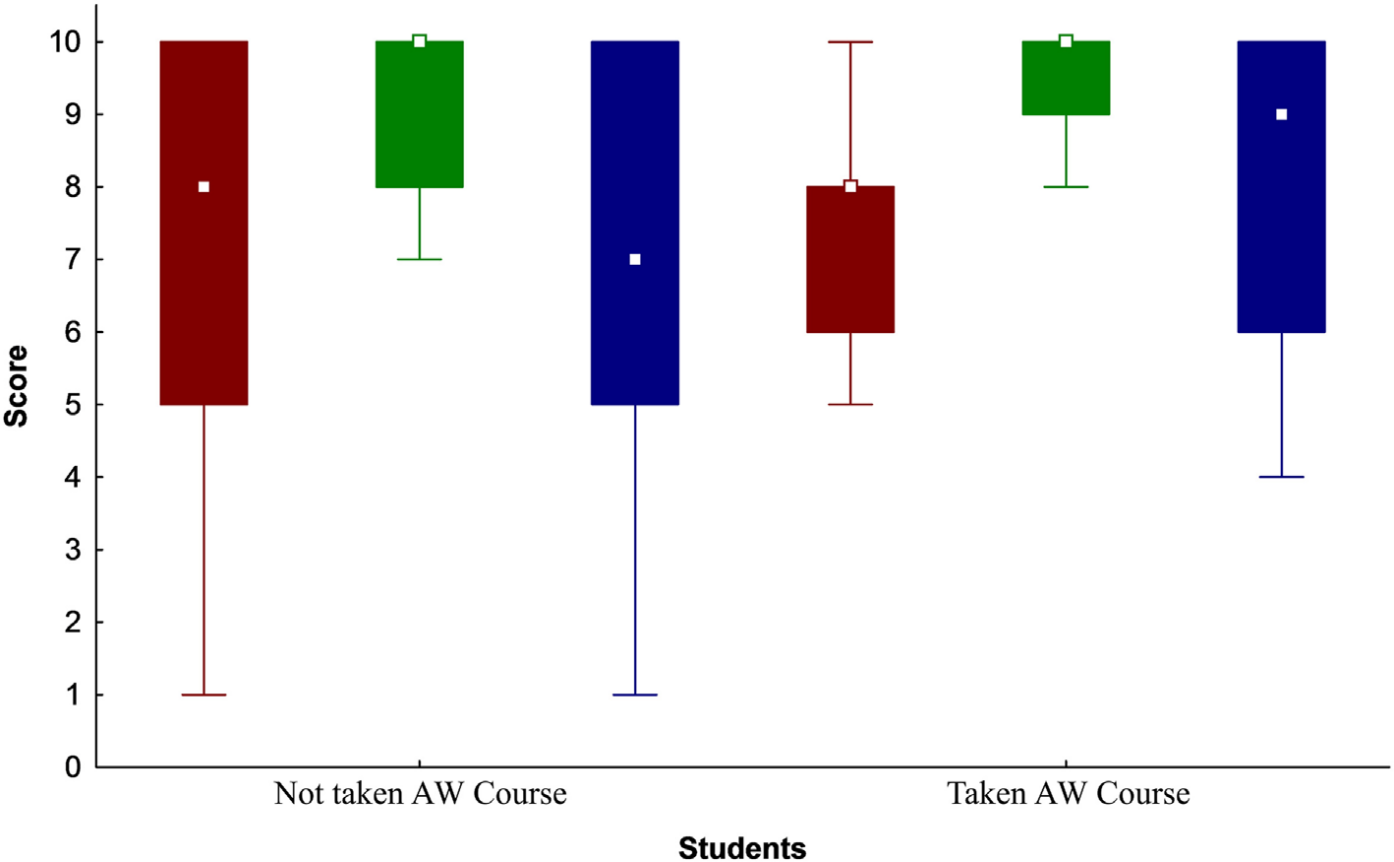
Students’ opinions regarding the influence level (in scores on the *Y* axis) of Animal Welfare (AW) on Human (red), Animal (green), and Environmental (blue) Health, groups according to whether they had taken the AW course (*n* = 15 for both groups) during their undergraduate studies. Median □; 25–75%, box; non-outlier range, whisker.

The survey among students demonstrated that 83% were not familiar with the OH concept and that 56% of them were students who did not take the AW course. Regarding the influence score of the AW on human, animal, and environmental health, the students scored with the median score of 8 (range 1–10, Q1 = 6 and Q3 = 9), 10 (range 1–10, Q1 = 9 and Q3 = 10), and 8.5 (range 1–10, Q1 = 5 and Q3 = 10), respectively. There was no significant difference between answers of students regarding the AW influence on human, animal, and environmental health considering their participation in the AW course (Figure 5). The correlations between the student’s knowledge of AW (based on self-grading) and how they scored the influence of AW on human, animal, and environmental health were 0.14, 0.42, and 0.57, respectively. Similar to the stakeholder’s survey, over 93% of the students stated that cooperation with the AWC can contribute to an improvement in human, animal, and environmental health.

### Transdisciplinary Evaluation

**Figure 6:**
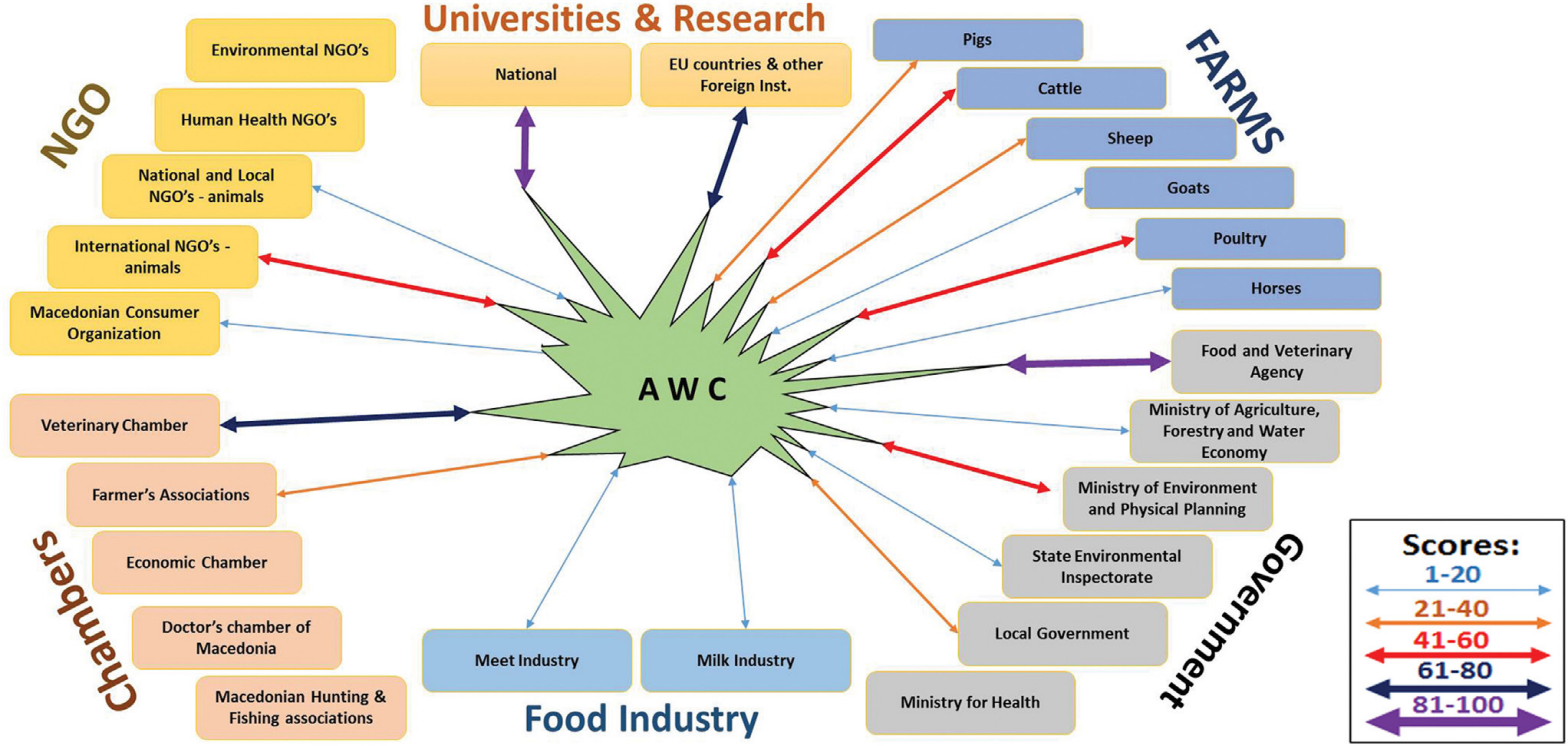
Transdisciplinary evaluation of the Animal Welfare Center (AWC), according to the CACA scoring system. Scores for the links between AWC and the actors in the six major groups of stakeholders are represented by different colors and arrow’s weights (legend on the right), while the absence of the arrows means no established cooperation. The shape of the AWC in the model corresponds to the level of cooperation of the AWC, i.e., the higher the spikes of the AWC shape, the higher level of cooperation with the actor.

During the identification phase, 27 actors who have direct relation to human, animal, or environmental health were identified. Considering the six major stakeholders’ groups, the distribution of the identified actors was: five actors in NGOs, six actors in the group Animal Farms, six actors/institutions from the Government, two in Universities and Research; six in the group called Chambers; and two actors from Food Industry. In the data collection phase, 18 different types of cooperation, with over 100 realized activities were found between the AWC and the identified actors. By using the CACA scoring system, the transdisciplinarity of the AWC from the OH perspective is presented in Figure 6.

## Discussion

The evaluation of the AWC from the OH perspective performed in this study demonstrates that the AWC, and consequently AW in general, has had an impact and contributes to improvement and securing not only animal health but also human and environmental health. The evaluations for OH initiatives performed in this case, mainly guided by the “A Handbook for evaluation of one health,” Chapter 3—A One Health Evaluation Framework (COST Action TD1404, NEOH), presents the role of AW in the OH concept and hypothesizes that the AW initiatives can also be seen as OH initiatives.

One Health-ness as a sum of characteristics that define integrated approaches to health (17), in this case, the sum of five dimensions, was used for representing the strengths and weaknesses of the AWC from the OH perspective. The assessment of the OH-ness performed on the AWC found several remarks and challenges of the method used. These are highlighted in red in the S1_OH-ness Scoring of the AWC in Supplementary Material. However, the NEOH Handbook for evaluation of OH has been developed further than the state at which it was used in this study. In this context, the number of dimensions representing the OH-ness (Figure 1) requires further discussions and analyses in the forthcoming studies. Finally, more widely practical usage of the OH-ness assessment inevitably will lead to its improvement and precision. For determining the effects of the AWC on OH as a secondary (indirect) impact, the terminology established in the paper by Rüegg et al. (17), the impact evaluation of the AWC was performed. This was accomplished by summarizing the opinions of different stakeholders and students about the AWC’s work and its relationship with OH (human, animal, and environmental health). However, the number of respondents participating in the impact evaluation was low for very detailed impact analysis.

The transdisciplinarity, as a vital part of the OH approach, was the core aspect for evaluation of the AWC from OH perspective. Rosenfield (18) sees transdisciplinarity as a help in health research by providing a holistic approach where the researchers will work with different stakeholders for the purpose of addressing a common problem. In addition, there was a consensus among research articles that transdisciplinarity is necessary for solving human–animal–environmental health issues (19). Summarizing the scores from the four pillars in the CACA scoring system gives an overview of the link’s strength between AWC and the stakeholders in society. However, the method for identifying the stakeholders and actors in this study may introduce some bias in the final results. Nevertheless, modeling the established links in the overall network of actors and stakeholders offers an overview of the transdisciplinarity of the AWC from the OH perspective. This also raised the expectations in this study for finding the place of the AW in OH. More broadly, since the AWC is an AW initiative, this could be perceived as an opportunity to determine where the AW stands in society from the OH perspective.

Improvements in AW and raising AW standards in the system were found as a major driver of the AWC. In fact, AW in general is recognized as a “complex, multi-faceted public policy issue which includes important scientific, ethical, economic and political dimensions” (20). This inevitably leads to a higher score for OH thinking for almost all AW initiatives. The AWC was a pioneer for acceptance and understanding the AW field in society, leading to a low match of the AWC with the environment, i.e., low scores within OH thinking. The same findings were reported for the OH concept. Familiarity with the OH concept among stakeholders in society and undergraduate students emphasized the need for higher involvement of OH in the undergraduate curriculum for veterinary studies and overall promotion of the OH concept in Macedonian society. The greatest familiarity with the OH concept was among stakeholders who had cooperation with the AWC, indicating that the group that was interested in AW, also has knowledge and/or interest in OH. This additionally supports the higher score for the AWC’s OH thinking.

The AWC OH planning and working were also scored highly. Higher dominant scores were the societal and broadness as one of the features of AW in general. OH working dimension includes transdisciplinarity and leadership of the AWC, also confirmed by impact and transdisciplinary evaluation within this study. The stakeholder’s scoring of the AWC influence on OH was highly related with the work’s perspective, knowledge, and information about the scope/work of the AWC. Thus, in the scoring for the human health, the farmers gave higher scores as the AWC was promoting the links between AW and prevention of diseases (including zoonoses) and food safety. The present knowledge from Academia about AW contributes to the maximum scores from this stakeholder for the AWC influence on animal health. The higher scores for environmental health given by the government resulted from the previous collaboration with the AWC on measuring the farm’s emissions to the environment. Both groups (with and without cooperation with the AWC) express their beliefs that they can contribute in improvement of human, animal, and environmental health through AWC, confirming the impact of the AWC on the OH concept. The responder’s request for improvements of the AWC from the OH perspective in all disciplines is actually supporting the transdisciplinarity approach and research in OH (21).

Transdisciplinary evaluation reveals wide spread of the AWC between different actors and stakeholders, presenting a relatively high level of overall transdisciplinarity. The shape of the AWC in Figure 6 indicates the presence of transdisciplinary work of the AWC with higher focus on the Universities and Research Institutions and Government, and low links with the NGOs and Chambers. Considering its main objective and primary work, the strongest link inevitably were the Universities and Research institutions. Since the AWC performs welfare training, assessments, and has an advisory role for the farmers, this group was also strongly linked with the AWC, with a room for improvement. The strongest link among governmental institutions was with the Food and Veterinary Agency as the authority responsible for AW legislation implementation in the country. The next strong link in this group was found with the Ministry of Environment and Physical planning, as a result of the AWC’s work on environmental measurements. There was no link between the Ministry of Health and also any other actors related to Human Health. However, human health is mainly related to the AW through the food safety and disease prevention (12, 13) which opens the doors for future close cooperation. The links with the Food Industry were present but showed a low score, due to the fact that AW training was the only activity provided. This custom designed model developed for the AWC transdisciplinarity can also be used for any other AW initiatives and would probably result in different findings of the strengths of various links. Regardless of these strengths, it is highly probable that any other AW initiative will also be widely spread in society, due to the transdisciplinary nature of the AW research and implementation.

The serious impact for a low score in OH learning was the lack of willingness of implementation of new AW standards by the society, mostly due to traditional farming practices, economic reasons, and poor awareness of these standards. This leads to very low learning focused on questioning, correcting, or improving existing practices and encouragement to see beyond the existing situation. On the other side, the impact of the AW course provided by the AWC for the students contributes to more consensual thinking about the AW influence on the three types of health. This was especially evident for animal and environmental health, where the AW knowledge had a direct effect in increasing the influence score of AW. The OH sharing score was due to the small amount of resources allocated for data sharing, the absence of procedures and mechanisms within the AWC for instant and easy information access to the stakeholders. This was also confirmed by the impact evaluation, where promotion of the AWC’s activities in society was mostly suggested. The OHI and especially OHR clearly indicates domination of the AWC “Operation” versus the “Infrastructure.” These results suggest that improvements and emphasis should be made in data sharing at the AWC, and further raising awareness in society for the AW standards, while the transdisciplinary evaluation gave the directions of future transdisciplinary work of the AWC.

The OH-ness of the AWC suggests that AW initiatives bear their own OH-ness, which can be scored and evaluated, like OH initiatives from all other disciplines. Stakeholders and students consider that the influence of AW on human, animal, and environmental health is relatively high (in the upper third of the scale) by setting the AW influence in the following order: 1. animal health; 2. environmental health; and 3. human health, with higher emphasis on animal health. The same was applied for the AWC influence confirming similar contribution by the AWC to OH as the AW topic itself. In addition, the AW transdisciplinarity from an OH perspective defined the AW place in the societal OH network. These results should be observed as relative findings that can vary when applying different evaluation methods or different AW initiatives. What is unquestionable is the evident impact of the AW to animal, environmental, and human health. We strongly believe that the AWC is not a unique case and that any other AW initiatives intentionally or unintentionally have an impact on OH and should be seen and evaluated as OH initiatives.

## Author Contributions

MR collected, analyzed the data, and drafted the manuscript. VI collected the data and revised the manuscript. MR and VI contributed to the study design and interpretation of the results. HO and DM reviewed, interpreted, and edited the manuscript.

## Conflict of Interest Statement

The authors declare that the research was conducted in the absence of any commercial or financial relationships that could be construed as a potential conflict of interest.

## Footnotes

^1^http://www.izsler.it/izs_bs/s2magazine/index1.jsp?idPagina=408

^2^https://www.fsai.ie/enforcement_audit/monitoring/national_official_labs.html

^3^http://neoh.onehealthglobal.net/
