# Supplementary material for: Positioning Animal Welfare in the One Health Concept through Evaluation of an Animal Welfare Center in Skopje, Macedonia
Source: Front Vet Sci. 2018 Jan 10;4:238. doi: 10.3389/fvets.2017.00238 (PMC5767597; doi:10.3389/fvets.2017.00238)
Supplement: Supplementary file 1 [file Table_1.DOCX]

**Positioning Animal welfare in the One Health concept through evaluation of an Animal Welfare Center in Skopje, Macedonia**

**Miroslav Radeski*, Helen O’Shea, Daniele De Meneghi, Vlatko Ilieski**

*** Correspondence:** Miroslav Radeski: miro@fvm.ukim.edu.mk

**OH-ness SCORING OF ANIMAL WELFARE CENTER**

**OH Thinking**

| No | Question | Explaination | Scoring | Answer |
| --- | --- | --- | --- | --- |
| Q1A | Which dimensions are considered by the initiative? | Please tick the relevant dimensions and add further dimensions as necessary. Use the explanations above to identify dimensions. | No scoring | □ Space – R. Macedonia; Municipalities; Farms  □ Time – Years  □ Dimension of Life population (animals – farm animals; stray animals; pets and laboratory animals)  □ Network/ Organisation – All levels (inter-sectorioal, inter-organizational; organization; working group)  □ Economy (National, Local, Farm, Houshold)  □ Legislation – EU, National and Local Law  □ Governance – EU DG Sante and National Government (National and Local law)  □ Values – EU and Public values for animal welfare  □ Research – International and National research activites and projects |
| Q1B | What importance is given to the different dimensions within the initiative? | This questions is to assess how important each dimension is within the concept and plan of the initiative. | 0 not considered  0.2 passive recognition  0.4 considered  0.6 weak involvement  0.8 strong involvement  1 essential | Space 0.4 __  Time 0.4 __  Dimension of Life 0.8 __  Network/ Organisation 0.8 __  Economy 0.2 __  Legislation 1 __  Governance 1 __  Values 0.6  Research 0.8 |
| Q1C | How balanced is the consideration of the different dimensions by the initiative | $Score=1-\frac{number of different scores-1}{number of dimensions}$  $AWC Score=1-\frac{5-1}{9}=1-\frac{4}{9}=1-0.44=\boldsymbol{0.56}$ | |  |
| Q2A | Which dimensions are relevant for the initiative? | This question checks for the interplay of the initiative with the system in which it operates. Use the following two questions to verify if the dimension is relevant:   1. Is this dimension important for the initiative, or can it be excluded without effect on (the expected and un-expected outcomes of) the initiative? 2. Does the initiative have an effect on the dimension? | No scoring | □ Space  □ Time  □ Dimension of Life  □ Network/ Organisation  □ Economy  □ Legislation  □ Governance  □ Values  □ Research |
| Q2B | How strongly would it affect the outcomes of the initiative, if the dimension was excluded? | The question checks for the importance of the dimensions to capture expected (and as far as possible unexpected) outcomes of the initiative. Please score the relevance of the dimension for the initiative. | 0 no relevance  0.2 slight relevance  0.4 some relevance  0.6 relevant  0.8 high relevance  1 essential | Space 0.2 __  Time 0.6 __  Dimension of Life 1 __  Network/ Organisation 1 __  Economy 0.6 __  Legislation 0.8 __  Governance 1 __  Values 0.4  Research 0.2  __  _____________________ __ |
| Q2C | How strong is the effect of the different dimensions on the initiative? | This question checks on the effect of the various dimensions on the initiative. Please score the importance of the influence of the dimension on the initiative. | 0 no relevance  0.2 slight relevance 0.4 some relevance  0.6 relevant  0.8 high relevance  1 essential | Space 0.8 __  Time 0.8 __  Dimension of Life 1 __  Network/ Organisation 1 __  Economy 0.8 __  Legislation 1 __  Governance 1  Values 0.8  Research 0.2  __  _________________________  _________________________  _________________________ |
| Q2D | How well does the consideration of dimensions by the initiative match the dimensions that it affects or is affected by? | This question aims at scoring the match between the initiative and the system within which it operates. Please consider the scores from Q1B and Q2B and Q2C to assess the match and provide a justification for scoring. | 0 no match  0.2 very weak match  0.4 weak match  0.6 match  0.8 strong match  1 perfect match  **WHAT DO YOU MEAN BY MATCH?!!!** | Space **0.2** __  Justification: Q1B=0.4 Q2B=0.2 and Q2C=0.8 |
|  |  |  |  | Time **0.4** __  Justification: Q1B=0.4 Q2B=0.6 and Q2C=0.8 |
|  |  |  |  | Dimension of Life **0.8** __  Justification: Q1B=0.8 Q2B=1 and Q2C=1 |
|  |  |  |  | Network/ Organisation **0.8** __  Justification: Q1B=0.8 Q2B=1 and Q2C=1 |
|  |  |  |  | Economy **0** __  Justification: Q1B=0.2 Q2B=0.6 and Q2C=0.8 |
|  |  |  |  | Legislation **0.8** __  Justification: Q1B=1 Q2B=0.8 and Q2C=1 |
|  |  |  |  | Governance **1** __  Justification: Q1B=1 Q2B=1 and Q2C=1 |
|  |  |  |  | Other: Values **0.2** __  Justification: Q1B=0.6 Q2B=0.4 and Q2C=0.8 |
|  |  |  |  | Other: Research **0.4** __  Justification: Q1B=0.8 Q2B=0.2 and Q2C=0.2 |
|  |  |  |  | Other: __  Justification: |
| Q2E | How well does the initiative match its environment? | This is the median score of Q2D | **0.4** |  |
| Q3A | How many scales are considered in the different dimensions of the initiative? | Name and count the scales or levels considered for each dimension. Refer to the examples above. |  | Space 3 Scales __  Scales: R. Macedonia; Municipalities; Farms |
|  |  |  |  | Time 1 Scales __  Scales: Years |
|  |  |  |  | Dimension of Life 1 Scales __  Scales: population (animals – farm animals; stray animals; pets and laboratory animals) |
|  |  |  |  | Network/ Organisation 5 Scales __  Scales: All levels (inter-sectorioal, inter-organizational; organization; working group, individuals) |
|  |  |  |  | Economy 4 Scales __  Scales: (National, Local, Farm, Houshold) |
|  |  |  |  | Legislation 3 Scales __  Scales: EU, National and Local Law |
|  |  |  |  | Governance 3 Scales __  Scales: EU DG Sante and National Governemnt (National) and Local Government |
|  |  |  |  | Values – 2 Scales  Scales: EU and National Public values for animal welfare |
|  |  |  |  | Research – 2 Scales  Scales: International and National research activites and projects |
|  |  |  |  | Other: __  Scales: |
|  |  |  |  | Other: __  Scales: |
| Q4 | How well do the number of dimensions and scales reflect an integrated approach to health? | This question aims at identifying whether the number of dimensions and the scales within those, which are considered in the approach are sufficient to consider the initiative an integrated approach. | 0 1 dimension, 1 scale  0.2 2-3 dimensions, 1 scale  0.4 1 dimension, >1 scales  0.6 2-3 dimensions, of which one >1 scales  0.8 2-3 dimensions, few at > 1 scales  1 very comprehensive | **1** |
| Q5 | What feature of the system is targeted by the initiative? | Determine what features of the system are targeted by the initiative: Events are singular, we can only react upon events. Patterns let us understand reality at a deeper level, i.e. trends, changes over time to which we can adapt. Thinking at structural level means thinking in terms of causal connections.  The key to lasting, high-leverage change on how the system operates. | - 1. events   0.6 patterns  1 structures | **1** Structures |
| Q6 | Where is the initiative situated in relation to the chain of events causing the problem and responding to it? | Initiatives can target different elements of the chain of events in relation to a problem. | 0 correcting damage  0.2 containing damage  0.4 preventing damage  0.6 avoiding the problem  0.8 redirecting the problem  1 modifying the socio-ecological system | **1** modifying the socio-ecological system – in terms of AW improvements and raising AW standards in the system as a whole |
| Q7 | How well does the initiative consider One Health and the 3 pillars of sustainability | Sustainability relies on the three pillars of society, environment and economy. One Health integrates the aspects of human, animal, plant and environmental health | 0 single pillar and aspect  0.2 one aspect 2 pillars  0.4 one aspect 3 pillars  0.6 two aspects 2 pillars  0.8 two aspects 3 pillars or 3 aspects 2 pillars  1 integrates 3 and more of each | **0.8** - 3 aspects (human, animal environment) and 2 pillars (society and economy) |
| System Thinking Assessment | | | | |
| Q8 | 1. What are the assumptions with which the initiative operates? To what degree does the initiative take into account the assessment of the validity of the underlying assumptions? (score) 2. Does the initiative foresee to assess the influence of the experiential history of the actors on the understanding of the system and the assumptions leading to the theory of change? Can this assessment change methods or gage involvement of new actors? 3. Is the initiative considering varying beliefs about evidence, values about health, cultural grounding as factors affecting the theory of change? 4. Does the project foresee assessing repeatedly how well the theory or model matches the system under study? 5. Is the initiative built along an iterative process? To what degree does the initiative foresee to revisit, assess and revise decisions and opinions iteratively. How clear is it outlined how the stakeholders will converge towards a common understanding of the system? 6. Are time delays recognised in the theory or model? Are they considered as part of the system? 7. Are mechanisms foreseen to correct the initiative throughout its implementation (self-assessment)? 8. Are leverage points identified or targeted in the system in order to induce change? 9. Are accumulation and depletion processes in the system described or addressed? 10. Are unintended consequences captured and can the initiative change accordingly if needed? 11. Are the most relevant dynamic feedbackloops of the system identified? 12. Is feedback used as a mechanism of action in the initiative? 13. Does the initiative identify subsystems and interactions between them? 14. Does the initiative consider appropriate time, space and resources for its aim, without neglecting an important system element? |  |  | **0.8 -** |
|  | **FINAL OH Thinking SCORE:***  ***** The final One Health thinking score is the mean score of all Questions. | \| **Q8** \| **0.8** \| \| --- \| --- \| \| **Q7** \| **0.8** \| \| **Q6** \| **1** \| \| **Q5** \| **1** \| \| **Q4** \| **1** \| \| **Q2E** \| **0.4** \| \| **Q1C** \| **0.56** \| |  | **0.79** |

**OH Planning**

| Task | Stakeholder | Responsibility | Authority  **It is not clear What “Authority” means???** | Means | Match |
| --- | --- | --- | --- | --- | --- |
| Training Workshops | Food and Veterinary Agency | Informing and Engagement | Linking with targeted groups | 1-2 person | 1 |
|  | farmers | Participation | Training | 1-2 person/farm | 1 |
|  | Food industry | Participation | Training | 1-2 person | 1 |
|  | Consumer Association | Participation | Training | 1-2 person | 1 |
|  | NGO’s | Participation | Training | 1-2 person | 0.5 |
|  | Local Governments | Participation | Training for stray dog’s | 5-6 persons | 0.5 |
| Legislation and EU standards implementation | Food and Veterinary Agency | Law enforcement | Consultancy | 1-2 person | 0.5 |
| Lab animals ethical committee | Food and Veterinary Agency | Law enforcement | Consultancy | 1-2 person | 0.5 |
|  | UKIM | Establishing committee | Consultancy and Participation | 5-6 persons | 0.5 |
| AW assessment | Farmers | Participation | AW assessment | Each farm | 1 |
| Emission Measurements from poultry and pig farms | Ministry of Environment and physical planning | Law enforcement | Measuring emissions from farms | 3 – 4 persons; instruments; farms | 1 |
|  | Poultry and Pig Farms | Law enforcement | Measuring emissions from farms | 3 – 4 persons; instruments; farms | 0.5 |
| AW Research | Universities and Higher Education | Cooperation | National and International | AW staff | 1 |
|  | Farmers | Research Implementation | Conducting research on farm animals | AW staff and farms | 0.5 |
| **OH Planning Score (mean of all matches in the table)** | | | | | **0.75** |

**OH WORKING** (there is no information how to calculate the total score for OH working and some of the questions are not quantifiable – need more work on this OH operation)

| No | Question | Explanation | Comments/Answers | | Scoring |
| --- | --- | --- | --- | --- | --- |
| Presentation of the societal problem within One Health | | | | |  |
| Q1 | What is the problem? Is the impact on society well described? | The question focuses on identification of the statement of the problem. | | **1** | Score the clarity of the problem statement on a scale *between* 0 (confused) and 1 (very clear). |
| Q2a | Which stakeholders are concerned by the problem? State the groups, individuals and dimensions that are concerned by the problem. | This question is intended to complement the chart in previous page and assess who is considered and involved. | |  |  |
| Q2b | Which stakeholders are involved in the initiative? State the groups, individuals and dimensions that are involved. |  | | **0.8** | If there is no governance and no involvement of other stakeholders concerned by the problem in the initiative, score =0; if there is governance but no involvement of other stakeholders concerned by the problem, score =0.2; if there is governance and involvement of other stakeholders concerned by the problem but those that have an influence on the problem are not involved, score =0.4; if few stakeholders concerned by/or with influence the problem are involved, score =0.6;if most stakeholders concerned by/or with influence are involved, score =0.8; if all stakeholders identified in the system are involved, score =1 |
|  | | | | | |
| No | Question | Explanation | | Comments/Answers | Scoring |
| Presentation of the societal problem within One Health | | | | |  |
| Q3a | What are the benefits of using TD rather than conventional approaches? | This question checks for the relevance of an integrated approach | |  |  |
| Q3b | Is transdisciplinarity (TD) required to solve this problem? |  | | **1** | Score the need for transdisciplinarity on a scale *between* 0 (not required) and 1 (cannot be achieved without). |
| Q4 | How does the approach/initiative compare to the concerned dimensions of the system? Compare Q2a to Q3a | This question aims at comparing the broadness of the approach to the extent of the problem. | | **0.8** | Score the correspondence on a scale from 0 to 1, with 0 no match and 1 very good match. |
| Q5 | Is the problem relevant to the health of people, animals, plants or the environment? | This question checks for the One Health Strategy | | **0.9** | Relevant to people/ animals/ plants or environment, score =0; relevant to any combination of two, score = 0.5; relevant to any combination of three, score =0.9, relevant to all, score =1 |

| Assessing broadness to further classify the initiative | | | | |
| --- | --- | --- | --- | --- |
| No | **Question** | **Explanation** | **Comments/Answers** | **Scoring** |
| Q6 | How diverse are the disciplines, methods, scales of analysis and/or social actors involved?  Please enumerate all disciplines, methods, dimensions and scales of analysis considered, as well as the social actors involved, as they were introduced in the section about OH thinking. | The question further distinguishes the purpose of a case study within the classes “fundamental understanding” and “problem solving”. A high diversity is typical for “comprehensive understanding” and “wicked problem solving”. | **1** | Score the diversity on a scale between 0 and 1, with 0 = very homogeneous project (e.g. only natural science, no interdisciplinarity, no participation: laboratory experiment), 0.5 intermediate diversity (e.g. two disciplines or two sectors, primarily natural or primarily social science, government agency and academia: disease surveillance in a food chain), 1 high diversity (e.g. participatory epidemiology, qualitative and quantitative approaches in natural and social science, integrated approach to develop a locally sustainable food system) |
| Q7 | To what extent is the non-scientific community involved? | This question aims at differentiating interdisciplinary from transdisciplinary projects | **0.6** | No, score = 0; community is consulted for problem definition, score 0.3; community is also consulted to develop solutions, score 0.6; community contributes to monitoring, implementation and/or decision-making, score =1. |
| Q8 | To what extent is the project/initiative inter-sectorial? | This question asks the reviewer to consider whether the case study applies cross-sectorial principles. | **1** | One sector involved, score 0; two sectors involved, score 0.3; three sectors involved, score 0.9; more sectors involved, score 1. |

|  | Assessing integration |  |  |  |
| --- | --- | --- | --- | --- |
| No | **Question** | **Explanation** | **Comments/Answers** | **Scoring** |
| Q9 | To what extent do the different disciplines work together? | The question probes for experience of interdisciplinary/transdisciplinary working. | **0.3** | 1. Are meetings with all disciplines (face-to-face or virtual) held frequently? 2. Are aims and objectives shared and clear to all? 3. Is there joint decision-making?   Score: 0= none of the above; 0.3=one of the above; 0.6=two of the above; 1=all of the above |
| Q10 | Are there power (i.e. academic or disciplinary dominance) or gender imbalances within the group, which risk biasing the process?   1. Across disciplines 2. Across sectors 3. Across ethnicities 4. Across social classes 5. Across gender 6. Other social difference…. please specify | This question probes for dominance of one discipline over the rest. | **Mean 0.85** | Power clustering, uneven power distribution, for each score 0; strong empowerment of all participants, score =1.   1. Across disciplines **0.75** 2. Across sectors **0.5 – mostly the AWC is dominating** 3. Across ethnicities **1** 4. Across social classes **1** 5. Across gender **1** 6. Other social difference…. please specify |
| Q11a | Are there cultural issues that need to be considered? If yes, kindly specify  (Issues may include mobbing, unequal task distribution based on ethnicity, racism, discrimination, paternalistic behaviour, etc. | This question looks for contexts which may inhibit the success | **1** | Score the effect of cultural issues on the project implementation  Many cultural issues effect project=score 0; moderate cultural issues effect project=score 0.4; few cultural issues effect project=score 0.8;No cultural issues involved that effect project=score 1. |
| Q11b | Are there religious issues that need to be considered? If yes, kindly specify  (Issues may include mobbing, unequal task distribution based on ethnicity, racism, discrimination, paternalistic behaviour, etc. | This question looks for contexts which may inhibit the success | **0.8 (religious slaughtering)** | Score the effect of religious issues on the project implementation  Many religious issues effect project=score 0; moderate religious issues effect project=score 0.4; few religious issues effect project=score 0.8;No religious issues involved that effect project=score 1. |
|  | **Assessing integration** |  |  |  |
| No | **Question** | **Explanation** | **Comments/Answers** | **Scoring** |
| Q12 | What is the spatial proximity among disciplines’ offices? | The question probes for readiness for collaboration. | **0.3** | Offices are on the same floor, score 1; offices are in the same building, score 0.8; offices are within walking distance, score 0.7; meetings require traveling up to 30 min, score 0.5; meetings can be held within a day, score 0.3; meetings require traveling > 1 day, score =0. |
| Q13 | Are there face-to-face interactions? If yes, how frequent are these interactions? |  | **0.4** (quarterly based meetings on an average) | Score the face-to-face interaction on a scale between 0 and 1, with for example no interaction, score =0; once a year, score = 0.2; monthly planned meetings, score 0.6; spontaneous frequent meetings, score 1. |
| Q14 | How innovative and how suitable is the combination of disciplines and fields of expertise for the specific purpose? | The question asks the reviewer to assess originality and suitability of the combination of disciplines and fields of expertise for the specific purpose. | **0.5** (there is an absence of direct involvement of human health sector) | No match, score =0; disciplines cover the defined problems, score 0.5; disciplines exceed the scope of the problem and allow unexpected findings, score 1. |
| Q15 | How balanced is the weaving of disciplines or fields of expertise? | The question assumes that an integration that balances disciplines or fields of expertise is a sign of high inter- and transdisciplinary quality. | **0.75** (the outcomes are mostly provided by the involved parties/disciplines) | Score the balance on a scale from 0 to 1, with 0 one discipline provides the outcomes, 1 a balanced combination of disciplines. |
| Q16a | Is a common One Health objective formulated that covers all the disciplines? | The question checks for One Health objectives in transdisciplinarity and for knowledge integration | **0.2** (despite the Animal Welfare, the One Health objective is not clearly stated) | Score the interdisciplinarity of the objective(s) on a scale from 0 to 1, with objectives for each discipline, score 0; common objective for all disciplines = 1. |
| Q16b | Can it serve in the process as a basis for knowledge integration? |  | **0.75** (Although the OH is not clearly stated, the knowledge integration is evident in the objectives) | Score the capacity of the objective to serve as a basis for knowledge integration on a scale from 0 to 1, with 0 no capacity, 1 very well suited. |

| Assessing reflection, learning and adaptation | | | | |
| --- | --- | --- | --- | --- |
| No | **Question** | **Explanation** | **Comments/Answers** | **Scoring** |
| Q17a | Is the approach to self-reflection, learning and adaptation *at individual, team and/or institutional level?* | The question assumes that planned stages of self-reflection and learning and the possibility to adapt the case study based on this is a sign of high inter- and transdisciplinary quality. | 0.6 | No self-reflection, learning and adaption, score =0; self-reflection at individual level, score 0.3; self-reflection, learning and adaption at individual and team level, score 0.6; self-reflection, learning and adaption at individual, team and institutional level, score 1. |
| Q17b | How likely is reflection going to feed back into corrective action within the case study? | The question asks the reviewer to assess whether the case study will connect reflection and action. | 1 | Score the likelihood on a scale from 0 to 1 with no feedback, score =0, feedback without action, score =0.3, feedback and defined action pathway, score =1. |
| Q18a | How flexible is the project design and timeline to respond to internal or external changes at short-term? | The question checks for feasibility of project design and timeline in the short-term. | 0.5 (the administration procedures and lack of human resources for short-term response) | Score the flexibility on a scale from 0 to 1, with 0 no flexibility, 1 high flexibility |
| Q18b | How flexible is the project design and timeline to respond to internal or external changes at mid-term? | The question checks for feasibility of project design and timeline in the medium term. | 1 | Score the flexibility on a scale from 0 to 1, with 0 no flexibility, 1 high flexibility |
| Q18c | How flexible is the project design and timeline to respond to internal or external changes at long-term? | The question checks for feasibility of project design and timeline in the long-term. | 1 | Score the flexibility on a scale from 0 to 1, with 0 no flexibility, 1 high flexibility |

| Assessing efficiency and effectiveness of the case study’s problem solving | | | | |
| --- | --- | --- | --- | --- |
| No | **Question** | **Explanation** | **Comments/Answers** | **Scoring** |
| Q19 | How elaborate is the problem and the initiative’s specific contribution to the problems solution? | The question assumes that an elaborated (complexity well-defined in detail) understanding of the problem and of the case study contribution to its solution is a sign of high inter- and transdisciplinary quality. | 0.75 (some of the partners are not clearly know the whole objectives of the AWC) | Score elaborate understanding of the problem on a scale from 0 to 1, with 0 not elaborate, 1 highly elaborate |
| Q20 | To what extent does the case study establish mechanisms for problem detection and solving beyond the current problem under investigation?” | The question asks the reviewer to assess whether the case study will support problem solving. | **0.5** (the AWC because of the lack of human resources is partly capable for problem detection) | Score the contribution of the present initiative to future problem solving on a scale from 0 to 1, with 0 no contribution, 1 establishment of sustainable and future oriented mechanisms. |

| Assessing management, social and leadership skills | | | | |
| --- | --- | --- | --- | --- |
| No | **Question** | **Explanation** | **Comments/Answers** | **Scoring** |
| Q21a | Describe the management structures involved in this initiative | The question gets a clear picture of management structures. | The Leader of the AWC and task leaders for different disciplines and projects |  |
| 21b | How well do the management structures match and support the initiative’s goal and combination of disciplines and fields of expertise? | The question assumes that an elaborated management structure is a sign of high inter- and transdisciplinary quality. | 0.75 | Score the aptitude of the management structure to support the goals and transdisciplinarity on a scale of 0 to 1, with 0 inappropriate structure, 1 well fit structure |
| Q22a | What is the type of leadership demonstrated in the case study? A) Small and collated (Single leader, central leader, informal connections, face-to-face processes, teambuilding, leader needs process skills B) Large and dispersed (Multiple leaders/champions, leaders in brokerage positions, coordination needed among leaders, leaders as translators and conflict handlers) |  | A |  |
| Q22b | How would you characterize the leadership in the initiative in regard to task-orientation, relationship-orientation and change-orientation |  | 0.5 | Score the leadership on a scale from 0 to 1 with 0 for no leadership, 0.1 a focus on task-orientation, 0.3 a focus on relationship-orientation, 0.3 a focus on change-orientation, 0.5 some of all, but unbalanced, 1 a well-balanced combination |
|  |  |  |  |  |

| Assessing management, social and leadership skills | | | | |
| --- | --- | --- | --- | --- |
| No | **Question** | **Explanation** | **Comments/Answers** | **Scoring** |
|  |  |  |  |  |
| Q23a | Does the initiative demonstrate open mindedness? | The question assumes that if the case study shows core values of inter- and transdisciplinary ethics, this is a sign of high inter- and transdisciplinary quality. | 1 | Score the open-mindedness on a scale from 0 to 1, with 0 self-centred atmosphere, 1 strong extroversion. |
|  | Does the initiative demonstrate self-reflection? | The question assumes that if the case study shows core values of inter- and transdisciplinary ethics, this is a sign of high inter- and transdisciplinary quality. | 0.25 | Score the self-reflection on a scale from 0 to 1, with 0 no self-reflection, 1 permanent thorough reflection |
| Q23b | Does the initiative demonstrate changing hierarchies? | The question assumes that if the case study shows core values of inter- and transdisciplinary ethics, this is a sign of high inter- and transdisciplinary quality. | 0.5 (some parts are static and the others are changing according the needs) | Score the change of hierarchies on a scale from 0 to 1, with 0 static hierarchy, 1 hierarchy adapts to situation at hand. |
| Q23c | Does the initiative demonstrate ability to bear and manage tensions? | The question assumes that if the case study shows core values of inter- and transdisciplinary ethics, this is a sign of high inter- and transdisciplinary quality. | 0.75 | Score the ability on a scale from 0 to 1, with 0 no ability, 1 high ability |

| Assessing team structure (well-structured vs. pseudo team) | | | | |
| --- | --- | --- | --- | --- |
| No | **Question** | **Explanation** | **Comments/Answers** | **Scoring** |
| Q24a | Is teamwork mentioned in this case study? | The question focuses on whether the case study is based on teamwork. The question assumes that if the case study shows teamwork across disciplines, this is a sign of high inter- and transdisciplinary quality. | 0.75 | Score the teamwork across disciplines on a scale from 0 to 1, with 0 no teamwork, 1 well-functioning teamwork. |
| Q24b | Describe any measures taken to encourage teamwork? | The question checks for practical measures that foster teamwork across disciplines | Different tasks and project are the initiations for creating different teams within the AWC |  |
| Q24c | How many teams are mentioned? | The question checks on the number of teams involved in the initiative: one, two, three or more teams related to this initiative? The question assumes that the more complex the case study, the more teams are present. | 1 | Please score on a scale from 0 to 1 with 0 no teamwork, 0.3=one team; 0.6=two teams; 1=three or more teams |
| Q24d | If more teams than one are mentioned, how good are the inter-team relations? | The question probes for how well different teams work together for the overall aim of the solving the problem. The question assumes that if the case study shows good inter-team relations, this is a sign of high inter- and transdisciplinary quality. | 0.5 (need for better cooperation between teams) | Please score the inter-team relations on a scale from 0 to 1 with 0 competition, 1 cooperation |
| Assessing team structure (well-structured vs. pseudo team) | | | | |
| No | **Question** | **Explanation** | **Comments/Answers** | **Scoring** |
|  |  |  |  |  |
| Q25a | Do all teams have clear objectives? | The question determines whether the team/s are well structured or not? | 0.75 | Score on a scale from 0 to 1, with 0 no clear objectives, 1 clear objective. For several teams use the average |
| Q25b | How closely do team members work together to achieve the team’s objectives? | The question determines whether the team/s are well structured or not? | 0.75 | Score on a scale from 0 to 1, with 0 no teamwork, 1=close collaboration. For several teams use the average. |
| Q25c | How clearly are the roles differentiated for team members within the team? | The question determines whether the team/s are well structured or not? | 0.5 (not always the team members have clear roles) | Score on a scale from 0 to 1, with 0 no clarity about roles, confusion; 1 clear roles. For several teams use the average |
| Q25d | Are/is the team(s) recognized by the community/department/s/official organizations as clearly defined team(s)? | The question determines whether the team/s are well structured or not? | 0.5 (mostly teams are working within the AWC, not as separate groups but the recognition of the team members is present) | Score on a scale from 0 to 1, with 0 no recognition, 1 recognition, appreciation and respect. For several teams use the average |
| Q25e | How frequently does the team(s) meet to discuss their effectiveness and how it could be improved? | The question determines whether the team/s are well structured or not? | 0 | Score the frequency on a scale from 0 to 1, with 0 never, 1 frequently |
| Q25f | How many people are there in the team/s? | The question determines whether the team/s are well structured or not? The question assumes that large teams of more than 15 are not well structured. | 1 | 2-5 people 🞎 6-9 people🞎 10-15 people🞎 more than 15 🞎  **SCORING IS MISSING** |

| Actors and competences | | | | |
| --- | --- | --- | --- | --- |
| No | **Question** | **Explanation** | **Comments/Answers** | **Scoring** |
|  |  |  |  |  |
| Q26a | How well do the disciplinary composition and the competence in the team permit the treatment of the essential aspects of their objective? | The question focuses on identification of transdisciplinarity in the team/s. | 0.75 (usually the selection of the team members is based on their competences for the specific objectives) | Score on a scale from 0 to 1, with 0 inappropriate team composition in terms of competence, 1 perfect match of competences and team roles. For several teams use the average |
| Q26b | Are the competences of the various disciplines appropriate to the problem and its solution (relevant knowledge, role in the case study, possibilities for implementing results)? | The question checks for competences of the different disciplines and whether these competences are relevant to the problem? | 0.75 | Score on a scale from 0 to 1, with 0 inappropriate team composition for the problem, 1 perfect match of team roles to solve problem efficiently and effectively. For several teams use the average |

| Problem formulation, focus, goals, and criteria of success | | | | |
| --- | --- | --- | --- | --- |
| No | **Question** | **Explanation** | **Comments/Answers** | **Scoring** |
| Q27a | How relevant is the initiative to One Health? | The question focuses on identification of the case study (problem) in terms of One Health strategy | 1 | Score on a scale from 0 to 1, with 0 not relevant, 1 highly relevant |
| Q27b | Is the One Health problem adequately translated into scientific questions? | The question checks the scientific questions raised, as well as probes for contribution to new knowledge | 0.5 (not so well translated since the AWC is mostly focusing on the Animal Welfare, not OH per se) | Score on a scale from 0 to 1, with 0 not well translated, 1 good translation |
| Q28a | Is the current state of knowledge taken into consideration? |  | 0.75 | Score on a scale from 0 to 1, with 0 not considered, 1 completely integrated |
| Q28b | Describe the innovation in relation to this state of knowledge. |  | Involvement of AW into OH concept |  |
| Q28c | Is this innovation relevant to One Health? |  | 1 | Score the relevance on a scale from 0 to 1 with 0 not relevant, 1 highly relevant |
| Q29 | How well do the methods envisioned, the interfaces of transdisciplinary collaboration, the form of integration in practice, and the outcome of the case study fit the solution strategy sought for in One Health? | The question checks for One Health objectives in transdisciplinarity and for knowledge integration | 0.5 | Score on a scale from 0 to 1 with 0 no fit, 1 complete fit |

| **OH Working Score (mean of all figures in the above questions)** | **0.70** |
| --- | --- |

**OH Learning**

**(Q1 socring is contradictory with Q2 and Q3 scoring – for our purposes we reversed the scoring in Q1 – in red), same cas with Q4 scoring with**

|  |  | | Scoring | | |
| --- | --- | --- | --- | --- | --- |
|  | INDIVIDUAL LEVEL | | | | |
| Q1 | No learning, only received information that may lead to learning, but have not practiced learning | | | 1 Always 0  0.75 Very Often 0.25  0.5 Sometimes 0.5  0.25 Rarely 0.75  **0 Never 1** | |
| Q2 | Adaptive learning - learning through which the obtained information are used to correct or improve procedures, existing competences, technologies and paradigms without necessarily examining or challenging the underlying beliefs and assumptions of the organization. | | | 1 Always  **0.75 Very Often**  0.5 Sometimes  0.25 Rarely  0 Never | |
| Q3 | Generative learning - learning that involved the modification of the organization’s underlying norms, policies and objectives that made me able to see beyond the situation and questioning operating norms. | | | 1 Always  0.75 Very Often  **0.5 Sometimes**  0.25 Rarely  0 Never | |
|  | TEAM LEVEL | | | | |
| Q4 | The teams only meet and exchange information for mere reporting purpose with no goal to support learning | | | 1 Always 0  **0.75 Very Often 0.25**  0.5 Sometimes 0.5  0.25 Rarely 0.75  0 Never 1 | |
| Q5 | In the teams different views are presented and defended and there is a search for the best view to support decisions that must be made at this time | | | 1 Always  0.75 Very Often  **0.5 Sometimes**  0.25 Rarely  0 Never | |
| Q6 | In the teams complex issues are explored by presenting different views as a means toward discovering a new view. The assumptions the team members have are presented and examined. | | | 1 Always  0.75 Very Often  0.5 Sometimes  **0.25 Rarely**  0 Never | |
|  |  | Scoring | | |  |
| ORGANISATIONAL LEVEL |  |  |  |  |  |
| Q7 | The existing information and knowledge that is circulating is collected | 1 Always  **0.75 Very Often**  0.5 Sometimes  0.25 Rarely  0 Never | | |  |
| Q8 | The existing information and knowledge that is circulating is stored | **1 Always**  0.75 Very Often  0.5 Sometimes  0.25 Rarely  0 Never | | |  |
| Q9 | The collected information and knowledge is made available to teams and individuals in various ways | 1 Always  0.75 Very Often  **0.5 Sometimes**  0.25 Rarely  0 Never | | |  |
| DIRECT ENVIRONMENT |  |  |  |  |  |
| Q10 | The direct environment in which the OH project/ initiative took place our is supportive for adaptive learning - learning that focuses on correcting or improving existing procedures, processes, competences and technologies | 1 Always  0.75 Very Often  **0.5 Sometimes**  0.25 Rarely  0 Never | | |  |
| Q11 | The direct environment in which the OH project/ initiative took place our is supportive for transformative learning - learning that focuses on questioning the existing norms and that encourages to see beyond the existing situation | 1 Always  0.75 Very Often  0.5 Sometimes  **0.25 Rarely**  0 Never | | |  |
| GENERAL ENVIRONMENT |  |  |  |  |  |
| Q12 | The general environment in which the OH project/ initiative took place our is supportive for adaptive learning - learning that focuses on correcting or improving existing procedures, processes, competences and technologies | 1 Always  0.75 Very Often  0.5 Sometimes  **0.25 Rarely**  0 Never | | |  |
| Q13 | The general environment in which the OH project/ initiative took place our is supportive for transformative learning - learning that focuses on questioning the existing norms and that encourages to see beyond the existing situation | 1 Always  0.75 Very Often  0.5 Sometimes  0.25 Rarely  **0 Never** | | |  |

| **Learning Level** | **Mean Score** |
| --- | --- |
| Individual Level | 0.75 |
| Team Level | 0.33 |
| Organizational level | 0.75 |
| Direct Envrionment | 0.38 |
| General Environment | 0.13 |
| **OH Learning Score (mean of all scores at all levels)** | **0.47** |

**OH Sharing**

(not explicit explanation how to combine all the scores for each question)

| No. | Evaluation elements | Short element description | Relevant in this initiative? | Describe assessment points | Qualitative assessment (describe) | Quantitative assessment  (i.e. 0-100%) |
| --- | --- | --- | --- | --- | --- | --- |
|  | (Name of the element) | (Description and references, if needed) | (Used for normalisation of overall score. E.g. if not using data, then variable description is not necessary and would therefore get 0, but if data used then variable description needed = 1) | (E.g. almost all variables in the databases used in the initiative are described carefully in English, ensuring a common understanding of what they mean or were derived from raw data) | (E.g. non-existent, very low level, poor, good, high level, excellent depending on what is being evaluated) | (Estimate on a scale from 0 (non-existing implementation/coverage of element) to 100% (full implementation/coverage of element) |
| 0 | Example: Stakeholder involvement | Are essential stakeholders involved at an appropriate level? | 1 | Essential stakeholders are engaged in most workpackages where appropriate and annual status reports and plenary discussion meetings ensure good stakeholder feedback mechanisms | High level of stakeholder involvement | 90 |
| 1 | Stakeholder identification process | Has a process to identify and involve all essential stakeholders (including governmental, academia, industry, NGOs) been described and followed in the initiative? | 1 | For each separate activity of the AWC the relevant stakeholders are identified and involved. | High level of stakeholder involvement for the relevant activity, but rarely the stakeholders are following the others, not relevant for them, AWC activities | 0.8 |
| 2 | Stakeholder involvement | Have essential stakeholders (including governmental, academia, industry, NGOs) been involved at an appropriate level throughout the duration of the initiative? | 1 | Each activity/project/initiative in the AWC is realized with the relevant stakeholders on different levels | Good level of stakeholder involvement | 0.7 |
| 3 | Internal information sharing mechanism | Does the initiative have appropriate mechanisms in place to facilitate sharing of information within the initiative? (E.g. newsletters, workshops, reports available to all, results getting published, online information sharing platform…...) | 1 | Mostly through workshops and papers, not exact mechanism is established within the AWC | Very low level – no sharing mechanisms is established | 0.2 |
| 4 | External information sharing mechanism | Does the initiative have appropriate mechanisms in place to facilitate sharing of information outside the initiative? (E.g. newsletters, workshops, reports available to all, results getting published, online information sharing platform…...) | 1 | Mostly through workshops and papers, not exact mechanism is established within the AWC | Very low level – no sharing mechanisms is established | 0.1 |
| 5 | Sharing resources | Have resources been allocated to ensure necessary data and information sharing? | 1 | No resources has been allocated | Non-existent | 0 |
| 6 | Data sharing agreement | Have appropriate (e.g. formal/written/signed) agreements been made concerning data sharing in the initiative? | 1 | Formal agreements are signed for sharing data with the partners regularly involved in the AWC | Poor level, because it’s based only with the regular partners on the AWC and is not widely accepted procedure | 0.4 |
| 7 | Data quality | Are mechanisms/procedures in place to ensure data quality, e.g. data completeness, error-checking and correction of errors, variable descriptions, description of aggregations/calculations, documentation available. | 1 | Data quality check is present for the data collected by the AWC | Excellent level, the lab within the AWC is accredited by International Standards for data collection. This is only for data collected by the AWC | 0.9 |
| 8 | Data storage | Are mechanisms/procedures in place to ensure safe and appropriate data storage? (e.g. type of software, server, backup) | 1 | There is a written procedure for data storage within the Laboratory of the AWC, but is not targeting the whole data of the AWC, No software present at the moment. | High level of data storage, but without existing software and wider written procedure for the all data of the AWC | 0.8 |
| 9 | Data accessibility | Are mechanisms/procedures in place to ensure safe and appropriate data accessibility to facilitate sharing? (e.g. is extraction of data feasible without access to experts, or are experts readily available for extraction of data, is the process of data extraction bureaucratic/ cumbersome/overly time-consuming?) | 1 | No procedures are present for this point | Non-existent | 0 |
| 10 | Data sharing | How well are data being shared between people within the initiative? (e.g. compartmentalised (score 0-33), shared between few groups (34-66), fully shared between all in the initiative (score 67-100). | 1 | The data are sheared with the team members, but rarely widely among all involved parties of the AWC | compartmentalised | 0.3 |
| 11 | Method sharing | How well are methods shared between people within the initiative?(e.g. compartmentalised (score 0-33), shared between few groups (34-66), fully shared between all in the initiative (score 67-100). | 1 | Sheared with the team members, but rarely widely among all involved parties of the AWC | compartmentalised | 0.3 |
| 12 | Results sharing | How are results shared between people within the initiative? (e.g. compartmentalised (score 0-33), shared between few groups (34-66), fully shared between all in the initiative (score 67-100). | 1 | Sheared between teams and relevant parties | shared between few groups | 0.5 |
| 13 | Institutional memory | How well does the initiative include the creation of potential institutional knowledge reservoirs for data, methods and/or results over time? | 1 | All the data, methods and results are owned by the Faculty (FVMS) but no regular and written procedure for institutional knowledge is present | Poor level | 0.2 |
| 14 | Resilience to change | Are mechanisms/procedures in place to safe-guard data and information access in case of system change, e.g. change of IT-system, data ownership, institutional organisations. | 1 | There is a written procedure for some of the AWC data (the Lab data) and the AWC staff is managing the data | High level of Resilience to change | 0.7 |
| 15 | Use of information in learning | To which extend are data and information produced in the initiative used for learning/education activities? | 1 | All the information are used and accessible for learning especially for students and at the training workshops | Excellent | 1.0 |
|  | **OH Sharing Score** |  | 15 questions) | (Mean score of all questions) |  | **0.46** |
