# Supplementary material for: Positioning Animal Welfare in the One Health Concept through Evaluation of an Animal Welfare Center in Skopje, Macedonia
Source: Front Vet Sci. 2018 Jan 10;4:238. doi: 10.3389/fvets.2017.00238 (PMC5767597; doi:10.3389/fvets.2017.00238)
Supplement: Supplementary file 2 [file Table_2.DOCX]

**Positioning Animal welfare in the One Health concept through evaluation of an Animal Welfare Center in Skopje, Macedonia**

**Miroslav Radeski*, Helen O’Shea, Daniele De Meneghi, Vlatko Ilieski**

*** Correspondence:** Miroslav Radeski: miro@fvm.ukim.edu.mk

***One Health Index and One Health Ratio***

***-extraction from “A handbook for evaluation of one health”, by Rüegg S, Häsler B, Zinsstag J. , Chapter 3-* A One Health Evaluation Framework, *, Draft version, November 2016***

## One Health Index and Ratio

Detailed derivation of the equation

OH Planning

Q

ScP

V

W

H

ScL

Learning

OH Thinking

ScT

ScS

ScTDD

TD & Leadership

Sharing

### One Health Index: the surface of the pentagon

The angle between two Sc is = 2Π/5

$$\sin\frac{2\pi}{5}=\frac{H}{ScT} ; H=ScT*\sin\frac{2\pi}{5}$$

$$triangle=\frac{ScP*H}{2}=\frac{ScP*ScT*\sin\frac{2\pi}{5}}{2}$$

$$Surface of Pentagon=\sum all triangles$$

$=\frac{\sin\frac{2\pi}{5}}{2}*\left\{ \left( ScP*ScT \right)+\left( ScL*ScP \right)+\left( ScS*ScL \right)+\left( ScTD*ScS \right)+\left( ScT*ScTD \right) \right\}$ (1)

### The One Health Ratio:

Surface of top left section

$$\sin\frac{\pi}{5}=\frac{Q}{ScP};\cos\frac{\pi}{5}=\frac{W}{ScP}$$

$$little triangle=\frac{Q*W}{2}=\frac{ScP*\sin\frac{\pi}{5}*ScP*\cos\frac{\pi}{5}}{2}=\frac{{ScP}^{2}*\sin\frac{\pi}{5}*\cos\frac{\pi}{5}}{2}=\frac{{ScP}^{2}*\sin\frac{2\pi}{5}}{4} \left\{ \sin2\alpha=2\sin\alpha\cos\alpha\right.$$

$$top left surface=\frac{\sin\frac{2\pi}{5}}{2}*\left\{ \left( ScT*ScP \right)+\left( ScTD*ScT \right) \right\}+\frac{{ScP}^{2}*\sin\frac{2\pi}{5}}{4}$$

$top left surface=\frac{\sin\frac{2\pi}{5}}{2}*\left\{ \left( ScT*ScP \right)+\left( ScTD*ScT \right)+\frac{{ScP}^{2}}{2} \right\}$ (2)

Surface of bottom right section

The angle between W and ScL is = Π/5

$$\sin\frac{\pi}{5}=\frac{V}{ScL};\cos\frac{\pi}{5}=\frac{W}{ScL}$$

$$little triangle=\frac{V*W}{2}=\frac{ScL*\sin\frac{\pi}{5}*ScL*\cos\frac{\pi}{5}}{2}=\frac{{ScL}^{2}*\sin\frac{\pi}{5}*\cos\frac{\pi}{5}}{2}=\frac{{ScL}^{2}*\sin\frac{2\pi}{5}}{4} \left\{ \sin2\alpha=2\sin\alpha\cos\alpha\right.$$

$$lower right surface=\frac{\sin\frac{2\pi}{5}}{2}*\left\{ \left( ScS*ScL \right)+\left( ScTD*ScS \right) \right\}+\frac{{ScL}^{2}*\sin\frac{2\pi}{5}}{4}$$

$lower right surface=\frac{\sin\frac{2\pi}{5}}{2}*\left\{ \left( ScS*ScL \right)+\left( ScTD*ScS \right)+\frac{{ScL}^{2}}{2} \right\}$ (3)

Ratio

$OHR=\frac{\left( ScT*ScP \right)+\left( ScTD*ScT \right)+\frac{{ScP}^{2}}{2}}{\left( ScS*ScL \right)+\left( ScTD*ScS \right)+\frac{{ScL}^{2}}{2}}$ (4)
