# Supplementary material for: Positioning Animal Welfare in the One Health Concept through Evaluation of an Animal Welfare Center in Skopje, Macedonia
Source: Front Vet Sci. 2018 Jan 10;4:238. doi: 10.3389/fvets.2017.00238 (PMC5767597; doi:10.3389/fvets.2017.00238)
Supplement: Supplementary file 3 [file Table_3.DOCX]

**Positioning Animal welfare in the One Health concept through evaluation of an Animal Welfare Center in Skopje, Macedonia**

**Miroslav Radeski*, Helen O’Shea, Daniele De Meneghi, Vlatko Ilieski**

*** Correspondence:** Miroslav Radeski: miro@fvm.ukim.edu.mk

***QUESTIONNAIRE FORM***

***Questionnaire for the work of the Animal Welfare Center from the One Health perspective***

The main objective of this questionnaire is to evaluate the work of the Animal Welfare Center from One Health perspective and to determine the possibilities for further improvements of the Center. Therefore your opinion is highly valued and will contribute for evaluation of the Center and for detecting the potential components for development. We are deeply grateful if you take small amount of your precious time and respond to the questions below.

Thank you in advance

The respondent is answering as a representative from:_______________________________

City:_______________________________

Gender:  Male  Female

1. *Questions for all respondents*
2. **How will you self-graded your knowledge about Animal Welfare?**

1 – no knowledge at all

2 – have minimal knowledge

3 – enough knowledge

4 – have good knowledge

5 – have excellent knowledge

1. **According to your opinion how big is the influence of Animal Welfare on Human Health?**

**1 2 3 4 5 6 7 8 9 10**

1. **According to your opinion how big is the influence of Animal Welfare on Animal Health?**

**1 2 3 4 5 6 7 8 9 10**

1. **According to your opinion how big is the influence of Animal Welfare on Environment?**

**1 2 3 4 5 6 7 8 9 10**

1. **Are you familiar with the “One Health” concept?**

- Yes
- No

1. **Until now did you established any kind of cooperation with the Animal Welfare Center?**

- Yes
- No

1. *Questions for the respondents that HAVE cooperation with the Animal Welfare Center*
2. **Please evaluate your cooperation with the Animal Welfare Center?**

1 – unsatisfied

2 – not so good

3 – good

4 – very good

5 – excellent

1. **On a scale from 1 – 10, what is the influence of the work of the Animal Welfare Center on Human Health?**

**1 2 3 4 5 6 7 8 9 10**

1. **On a scale from 1 – 10, what is the influence of the work of the Animal Welfare Center on Animal Health?**

**1 2 3 4 5 6 7 8 9 10**

1. **On a scale from 1 – 10, what is the influence of the work of the Animal Welfare Center on Environment?**

**1 2 3 4 5 6 7 8 9 10**

1. **Do you think that by your cooperation with the Animal Welfare Center you are contributing in improvement of the Human, Animal and Environmental Health?**

- Yes
- No

1. **In which areas the Animal Welfare Center should expand its work in order to improve the Human, Animal and Environmental Health?**

- Education
- Veterinary medicine
- Food and food processing
- Economy and marketing
- Environment
- Human medicine
- Legislation
- Civil sector
- Other__________________________

1. **In which areas you can cooperate with the Animal Welfare Center in order to improve the Human, Animal and Environmental Health?**

- Education
- Veterinary medicine
- Food and food processing
- Economy and marketing
- Environment
- Human medicine
- Legislation
- Civil sector
- Other__________________________

1. **Will you continue your cooperation with the Animal Welfare Center?**

- Yes
- No

1. **Please give any kind of comments and recommendations considering your cooperation with the Animal Welfare Center:**
2. *Questions for the respondents that DON’T HAVE cooperation with the Animal Welfare Center*
3. **Are you familiar with the work of the Animal Welfare Center?**

- Yes
- No

1. **Were you involved in any kind of organized activity/project in relation to Animal Welfare?**

- Yes (*please state how were you involved*)

__________________________________________________________________________________________________________________________________

- No

1. **Why you did not have cooperation with the Animal Welfare Center?**

**___________________________________________________________________________________________________________________________________________________________**

1. **Do you think that if you cooperate with the Animal Welfare Center you will contribute in improvement of the Animal Welfare?**

- Yes
- No

*Please elaborate your answer:* ___________________________________________

________________________________________________________________________

1. **Do you think that if you cooperate with the Animal Welfare Center you will contribute in improvement of the Human, Animal and Environmental Health?**

- Yes
- No

1. **Please give your comments and recommendations for the Animal Welfare Center:**
2. *Respondent’s information (optional):*

- *If you agree you can write your contact information for further cooperation with the Animal Welfare Center:*

Name and Surname: ____________________

Institution/Organization:______________________

Phone:____________________

e-mail:______________________

**Thank you for your cooperation,**

**Animal Welfare Center**
